# First report of a Japanese family with spinocerebellar ataxia type 10: The second report from Asia after a report from China

**DOI:** 10.1371/journal.pone.0177955

**Published:** 2017-05-19

**Authors:** Hiroyuki Naito, Tetsuya Takahashi, Masaki Kamada, Hiroyuki Morino, Hiroyo Yoshino, Nobutaka Hattori, Hirofumi Maruyama, Hideshi Kawakami, Masayasu Matsumoto

**Affiliations:** 1 Department of Clinical Neuroscience and Therapeutics, Hiroshima University Graduate School of Biomedical and Health Sciences, Hiroshima, Japan; 2 Department of Neurological Intractable Disease Research, Kagawa University School of Medicine, Kagawa, Japan; 3 Department of Epidemiology, Research Institute for Radiation Biology and Medicine, Hiroshima University, Hiroshima, Japan; 4 Research Institute for Diseases of Old Age, Juntendo University School of Medicine, Tokyo, Japan; 5 Department of Neurology, Juntendo University School of Medicine, Tokyo, Japan; 6 Japan Community Health care Organization, Hoshigaoka Medical Center, Osaka, Japan; University of Texas MD Anderson Cancer Center, UNITED STATES

## Abstract

Spinocerebellar ataxia type 10 (SCA10) is an autosomal-dominant cerebellar ataxia that is variably accompanied by epilepsy and other neurological disorders. It is caused by an expansion of the ATTCT pentanucleotide repeat in intron 9 of the *ATXN10* gene. Until now, SCA10 was almost exclusively found in the American continents, while no cases had been identified in Japan. Here, we report the first case of an SCA10 family from Japan. The clinical manifestations in our cases were cerebellar ataxia accompanied by epilepsy, hyperreflexia and cognitive impairment. Although the primary pathology in SCA10 in humans is reportedly the loss of Purkinje cells, brain MRI revealed frontal lobe atrophy with white matter lesions. This pathology might be associated with cognitive dysfunction, indicating that the pathological process is not limited to the cerebellum. Examination of the SNPs surrounding the SCA10 locus in the proband showed the “C-expansion-G-G-C” haplotype, which is consistent with previously reported SCA10-positive individuals. This result was consistent with the findings that the SCA10 mutation may have occurred before the migration of Amerindians from East Asia to North America and the subsequent spread of their descendants throughout North and South America.

## Introduction

Spinocerebellar ataxia type 10 (SCA10; OMIM#603516) is an autosomal-dominant neurodegenerative disorder presenting with ataxia, seizure and other neurological symptoms [1]. SCA10 is caused by a large expansion of a pentanucleotide ATTCT repeat in intron 9 of the *ATXN10* gene [2]. The ATTCT repeat region in SCA10 ranges from 9 to 32 repeats in the normal population, but it may be as large as 4,500 repeats in alleles of affected persons [2,3]. The physiological function of the 55-kd ataxin-10 protein encoded by the *ATXN10* gene remains largely unknown [4].

Recent studies have shown that RNA toxicity is an underlying mechanism in diseases with repeat expansions in non-coding sequences, including such diseases as SCA8, SCA31, SCA36, Huntington disease-like 2 [5], myotonic dystrophy types 1 and 2, fragile X-associated tremor/ataxia syndrome, Fuchs endothelial corneal dystrophy [6] and amyotrophic lateral sclerosis and frontotemporal dementia *C9ORF72* expansion [7]. Similarly, the RNA-mediated gain of toxic function, but not the loss of function of ataxin-10, is considered to be a key pathogenic mechanism in SCA10 [8].

Currently, SCA10 has mainly been found in patients from American continents, and it is the second most common form of SCA in Mexico and Brazil, after SCA2 and SCA3, respectively [4]. Clinical manifestations in Mexican SCA10 families include cerebellar ataxia accompanied by epilepsy and other extracerebellar symptoms, including cognitive impairment and sensory polyneuropathy [9], while the most common phenotype in southern Brazil is pure cerebellar ataxia [10]. Haplotype analyses suggest a common ancestral origin of SCA10 in Mexican and Brazilian SCA10 families, which is generally considered to have arisen in an ancestral Amerindian population [11]. A population-based epidemiological study in Japan showed no reports of SCA10 cases [12]. However, an SCA10 family with epilepsy was recently reported in China, suggesting the possibility that the original SCA10 mutation may have occurred before the divergence of Proto-Amerindians from ancestral Asians [13]. In this report, we present the first known case of an SCA10 family from Japan, thereby proving a new clue about the geographical distribution of this disease.

## Materials and methods

### Ethics statement

The written informed consent used for this study was approved by the Human Subjects Committees of Hiroshima University. Blood samples were drawn, and genomic DNA was extracted from leucocytes using conventional procedures.

### Family history and clinical examination

A detailed history was taken, and a clinical examination and laboratory tests were performed.

### SCA10 expansion screening

DNA samples that had been digested by *Eco*RI were separated on a 0.8% agarose gel, transferred to a nitrocellulose membrane, and subsequently analyzed using Southern blotting to assess the sizes of SCA10 expansions [2]. The number of ATTCT repeats in intron 9 of the *ATXN10* gene was analyzed using polymerase chain reaction (PCR) amplification, using the primers forward (5'-GATGGCAGAATGATAAACTCAATCATGT-3') and reverse (5'-CCTGGGCAACATAGAGAGACTTCATC -3'). PCR was carried out as follows: 40ng of genomic DNA, 2.5μM of each primer, 2.5mM dNTPs, 1M BETAIN, 10X Ex Taq Burffer and Ex Taq Hot Start Version. The PCR conditions consisted of an initial denaturing at 98°C for 2 min, 30 PCR cycles (98°C for 10 s, 60°C for 1 min), and additional extension at 72°C for 7 min. Repeat-primed PCR was also conducted to verify the abnormal repeat expansion, using a forward primer (5'-GATGGCAGAATGATAAACTCAATCATGT -3'), a first reverse primer (5'-TACGCATCCCAGTTTGAGACGAGAATAGAATAGAATAGAATAGAATAGAATAGAATAGAATAGAAT-3'), and a second reverse primer (5'-TACGCATCCCAGTTTGAGACG-3'). Each PCR product was analyzed by 3130xl Genetic Analyzer (Life Technologies, Carlsbad, CA, USA) and the repeat number was calculated using following equation: Repeat number = ((size-118)/5) x 0.998–1.5271. Southern blotting, PCR and repeat-primed PCR were performed at BML, Inc. (Saitama, Japan).

### SCA10 haplotype analysis

SCA10 haplotype analysis of the proband and her mother was performed using PCR primers for single-nucleotide polymorphisms (SNPs). The PCR primers and conditions for these SNPs have been described previously [14]. The SNPs that flank the SCA10 expansion were examined in a previous study [14] and define the C (rs5764850)-expansion-G (rs72556348)-G (rs72556349)-C (rs72556350) haplotype. The PCR primers, amplification conditions and restriction fragment length polymorphisms were used as previously described [15].

### Genetic-associated frontotemporal lobar degeneration analysis

For direct sequence analysis, each exon was amplified by PCR using primers for *MAPT* and *PGRN*. These PCR products were analyzed by DNA sequence as previously described [16]. We performed repeat-primed PCR as previously described to assess an expansion of the noncoding GGGGCC hexanucleotide repeat in the *C9orf72* gene [17].

## Results

### History and clinical examination

**Fig 1:**
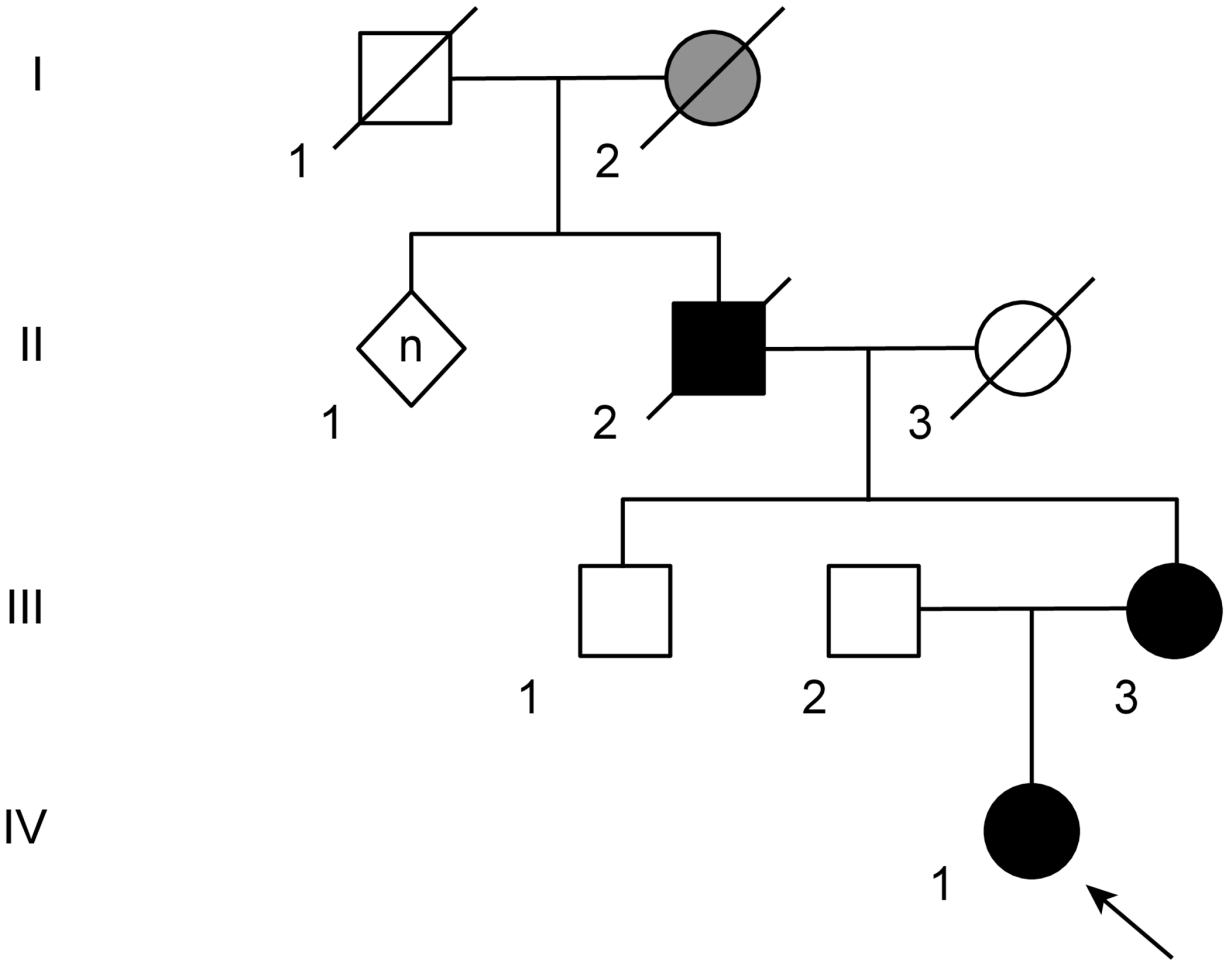
Pedigree chart of the Japanese spinocerebellar ataxia type 10 (SCA10) family. The proband (IV-1) and her mother (III-3) were genotyped and found to carry an expanded ATTCT region (approximately 1,500 repeats) in the SCA10 gene. Squares, males; circles, females; diamond, unknown gender; black fill, affected; gray fill, suspicious case; white fill, unaffected.

The family in this report originated from Shikoku Island, one of the four main islands of Japan, which is located south of Honshu and east of the island of Kyushu. All of the family members were of Japanese origin. The pedigree chart is depicted in Fig 1.

**Fig 2:**
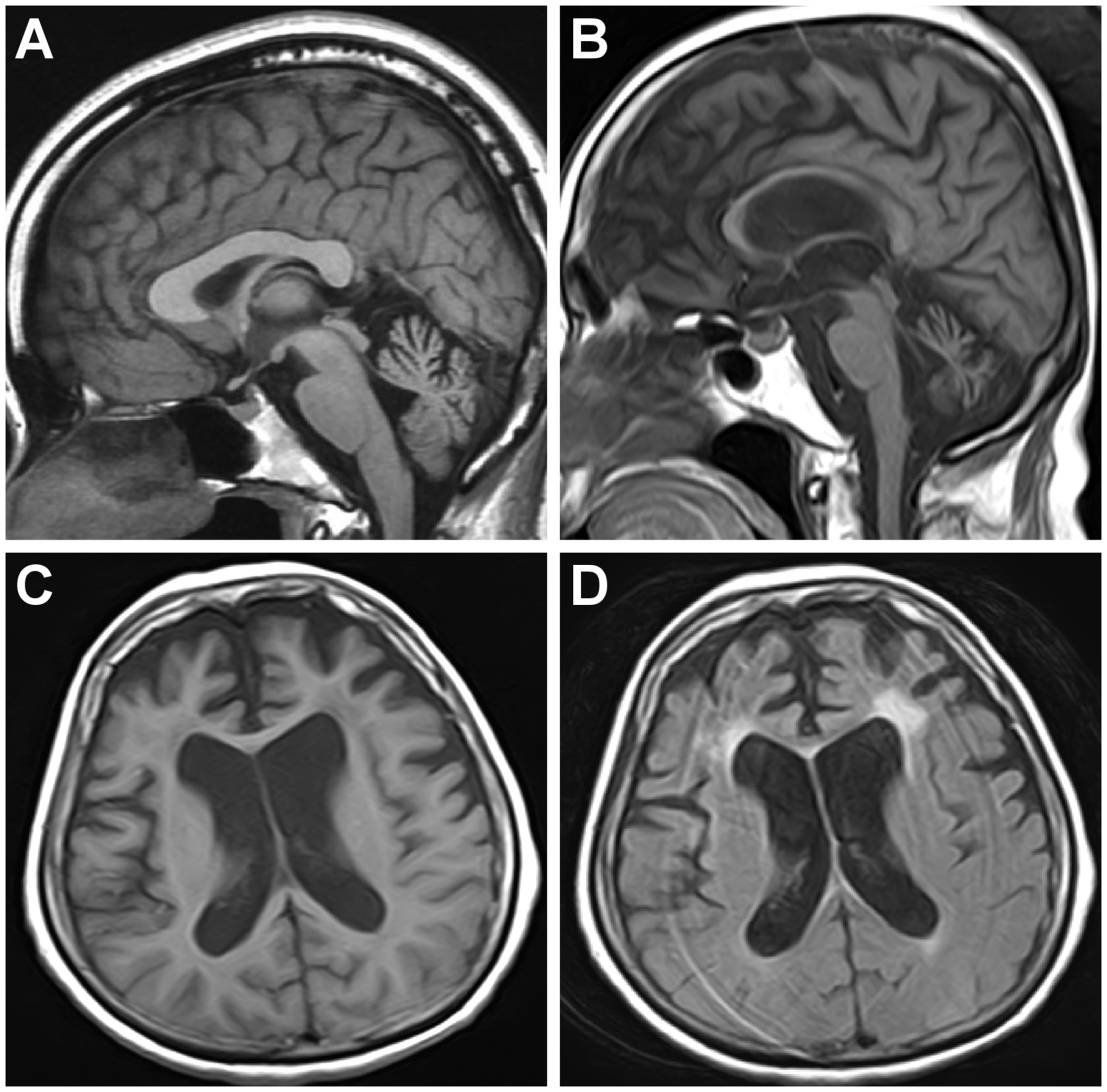
Brain MRI of the proband (IV-1) and her mother (III-3). T1WI sagittal brain MRI image showings cerebellar atrophy in the proband (IV-1, 39 years old) and her mother (III-3, 65 years old) (A, B). Additionally, T1WI and FLAIR axial images show frontal atrophy with white matter lesions in the mother (III-3) (C, D).

A 34-year-old Japanese woman (IV-1) presented to our hospital with progressive unsteadiness of gait. She had no significant medical history and was not taking any medications. Upon examination, she displayed horizontal nystagmus, ataxic dysarthria, mild left limb ataxia and a wide-based gait. Motor and sensory nerve functions were preserved, except for brisk tendon reflexes, and muscle tonus was normal. Blood tests, including a blood cell count, a general chemistry panel and thyroid function tests, were normal. She was initially diagnosed with hereditary cerebellar SCA because her mother also had a history of cerebellar ataxia. However, genetic testing for SCA1, SCA2, SCA3, SCA6 and DRPLA revealed no repeat expansions. Five years after her first visit, she experienced two episodes of complex partial seizures with alert mental status and automatism; they were effectively treated with levetiracetam. Brain MRI showed cerebellar atrophy (Fig 2A), and EEG showed bilateral epileptiform discharges over the frontal-parietal lobes. Seven years after the first visit, her score on the Scale for Assessment and Rating of Ataxia was 9. Additionally, neuropsychological tests (Montreal Cognitive Assessment score 28/30) showed normal cognitive function, and she had no difficulty working as a pharmacist.

The 65-year-old mother of the proband (III-3) first noticed tremor of both hands at 35 years of age, which was accompanied by gait imbalance and dysarthria. She began to have seizures accompanied by loss of consciousness at the age of 55 years. The frequency of her seizures spontaneously decreased after she reached 60 years of age. She also suffered from dysphagia, disorientation and cognitive impairment, for which she was prescribed donepezil. She is currently bedridden with an indwelling urinary catheter and has undergone gastrostomy. Her neurological examination revealed ataxic dysarthria and cognitive impairment with a preserved ability to follow simple instructions. Her physical examination revealed slow saccades with bilateral gaze-evoked nystagmus, dysphagia and prominent limb ataxia without pyramidal or extrapyramidal signs. She was unable to sit, stand or walk even with support. In addition to cerebellar atrophy, brain MRI showed atrophy of corpus callosum and of the bilateral frontal lobes with white matter lesions (Fig 2B–2D).

### Genetic testing

**Fig 3:**
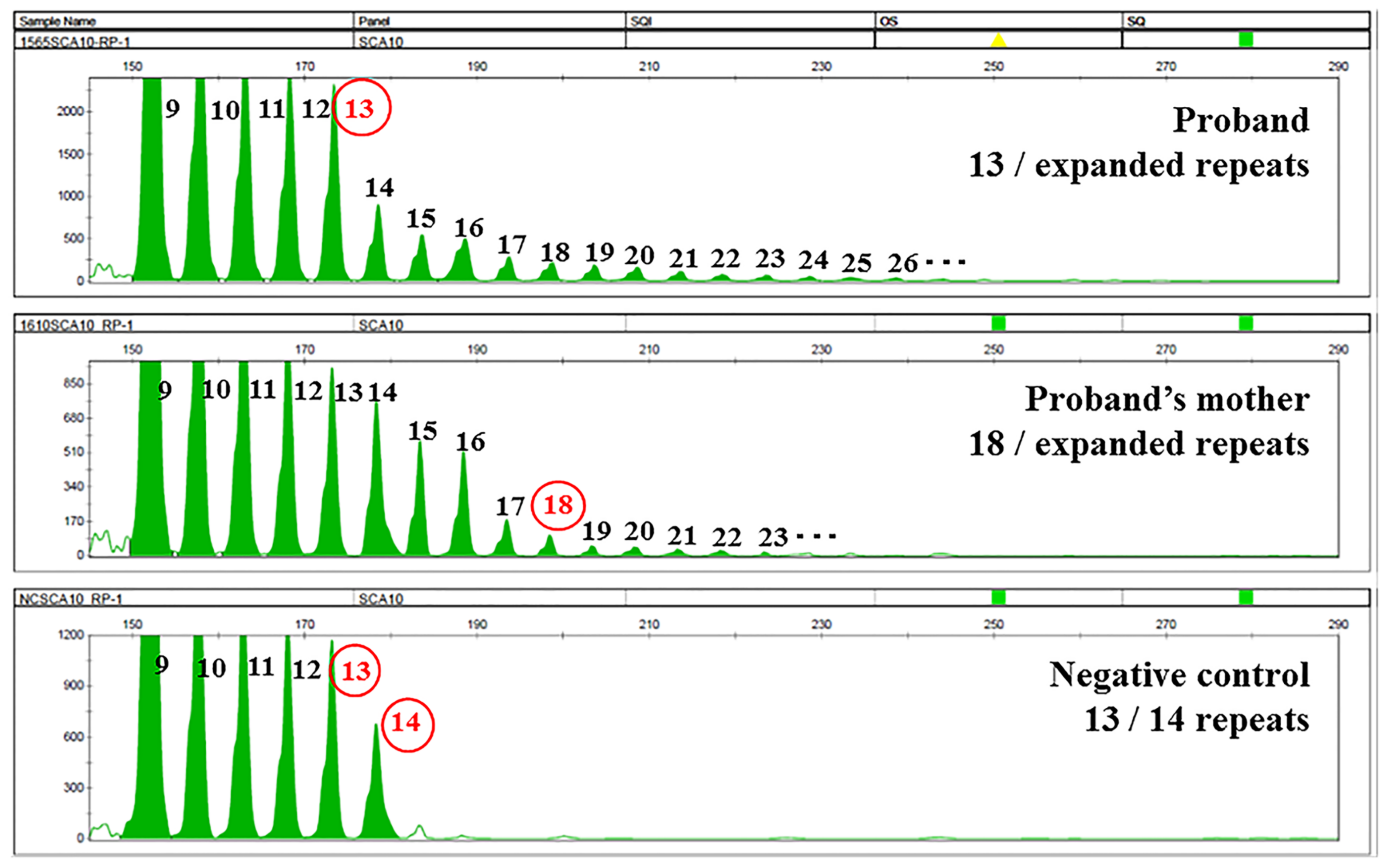
Fluorescent repeat-primed PCR analysis in SCA10. Fluorescent repeat-primed PCR analysis of the *ATXN10* gene revealed an expanded ATTCT pentanucleotide repeat in the proband (IV-1) and in her mother (III-3).

**Fig 4:**
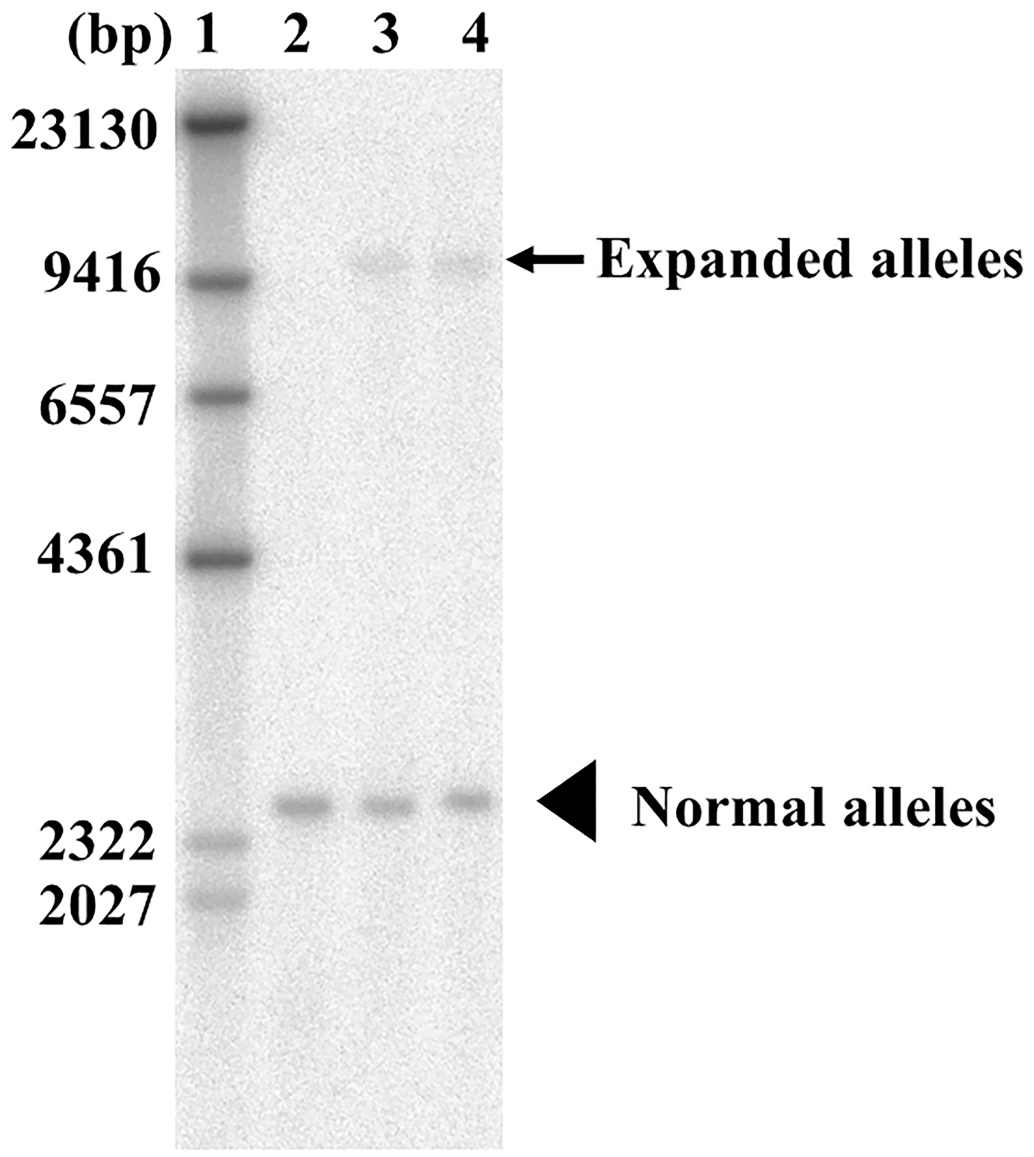
Southern blot analysis in SCA10. Lane 1 is a size marker, lane 2 is a negative control (13/14 repeats), and lanes 3 and 4 are DNA samples from the proband and her mother, respectively. Southern blot analysis shows an SCA10 repeat expansion of approximately 1,500 repeats in the proband (IV-1) and in her mother (III-3). The arrow shows expanded alleles and arrow head shows normal alleles.

DNA was extracted from peripheral blood leukocytes, and tests for SCA1, SCA2, SCA3, SCA6 and DRPLA were performed; all of the tests produced normal results. PCR showed a single band corresponding to 13 repeats in the proband and 18 repeats in her mother; the single bands implied that these repeat regions were either homozygous or hemizygous. Additionally, repeat-primed PCR analysis showed characteristic ladder products that were longer than 13 repeats in the proband and longer than 18 repeats in her mother (Fig 3). Southern blot analysis revealed an ATTCT repeat expansion of approximately 1,500 repeats in both cases and the expansion in the proband was only slightly larger than that of the proband’s mother (Fig 4). The haplotype of the proband (IV-1) is homozygous for the C (rs5764850)-expansion-G (rs72556348)-G (rs72556349)-C (rs72556350) haplotype, which is identical to a previously described genotype [11,13,14] (Table 1). One of the haplotype of the proband’s mother was also a common haplotype among SCA10 patients. The proband and her mother did not have *MAPT* and *PGRN* gene mutations, and repeat-primed PCR revealed no expanded hexanucleotide repeats in the *C9orf72* gene (S1 Fig).

**Table 1:** Haplotype analysis of single-nucleotide polymorphisms (SNPs) surrounding the SCA10 locus in the proband.

SNP ID	North American SCA10 [14]	Brazilian/Mexican SCA10 [14]	Japanese SCA10 (proband)	Japanese SCA10 (proband’s mother)
rs136002	A/G	A	A	A
rs5765626	G	G	G	G
rs5764850	C/A	C	C	C/A
**SCA10**				
rs72556348	G	G	G	G/A
rs72556349	G	G	G	G/A
rs72556350	C	C	C	C
rs136005	C/T	C	C	C
rs9614518	A	A	A	A
rs6006808	G	G	G	G
rs11912672	A	A	A	A
rs9614781	C	C	C	C

## Discussion

We report here the first Japanese family affected by SCA10, which is one of the increasing number of autosomal-dominant cerebellar ataxias [18]. The prevalence of different types of SCA differs substantially across the world. Machado-Joseph disease/SCA3, SCA6, SCA31 and DRPLA are reportedly common among autosomal- dominant SCA in Japan [12,19,20]. However, the relative regional frequencies of SCA also differ within Japan [20–22], likely reflecting the origin of individuals of differing ethnicities. Previous reports have indicated that approximately 10–30% of patients have an undetermined diagnosis among the relative frequencies of autosomal-dominant SCA in the Japanese population [18,19]. In nationwide investigations of autosomal-dominant SCA, no cases of SCA10 were identified in France, Italy, Portugal, China, Thailand or Japan [12,23–27].

SCA10 was first identified in families originating from Mexico [2,28] and was subsequently reported in families of Brazilian origin [10]. Until recently, SCA10 has been reported primary in North and South America, but cases of SCA10 reported outside the American continents have occurred in Europe in an immigrant from Peru [29] and in China [13]. To date, nearly all cases of SCA10 from Latin America have been identified in individuals having Amerindian ancestry [1,9–11,14]. Mexican and Brazilian families with SCA10 originating from Latin America shared the same intragenic haplotype, suggesting that SCA10 in Latin America has a common origin from an Amerindian population [11]. The report of a patient of Sioux Indian descent from North America indicates that the SCA10 mutation may have occurred early, when the Americas were first being populated [14]. Additionally, population and genomic data suggest that Amerindians and Native American ancestors migrated from East Asia across the Bering Strait land bridge to North America and that their descendants spread throughout North and South America [30,31]. Interestingly, Wang, et al. recently reported the presence of SCA10 in China of Han descents, and the haplotype was shared by persons from North America, Mexico, and Brazil, in agreement with this hypothesis [13]. The haplotype in our case was also identical to that in previous reports [11,13,14], further supporting the hypothesis. However, we cannot exclude the possibility that the backflow of human migration from America to East Asia through the Bering Strait [32] affected SCA10. In addition, each SNPs surrounding the SCA10 locus in our patients are relatively frequent among Japanese [33]. Therefore, there is another possibility that the shared haplotype in our case might also be frequent, as previously suggested by Wang, et al [13].

Our cases have several features in common with previous reports. First, extracerebellar signs, including epileptic seizures, mild pyramidal signs and cognitive dysfunction, were present in our case family. The extracerebellar signs associated with SCA10 are epileptic seizures, pyramidal signs, polyneuropathy and cognitive and neuropsychiatric impairment [9]. As SCA10 cases in Mexican families are often associated with extracerebellar signs; the clinical presentation in our case closely resembles that of Mexican patients with SCA10. Second, anticipation in our case family was not remarkable. Southern blotting showed that the repeat size in the proband was only slightly larger than that of her mother, suggesting that repeat size is relatively stable in case of maternal transmission [34]. Third, the difference in the age of onset of epileptic seizure between the two patients in our family was approximately 20 years, though the repeat sizes were slightly different between them.

Cognitive disability is more common in SCA10 with epilepsy than in that without epilepsy [13,35,36]. Whereas overt progressive dementia is not observed, some individuals with SCA10 exhibit mild cognitive dysfunction (IQ~70) and suffer from mood disorders, including depression and aggressive behavior [9]. However, neuroradiological and pathological examinations have not revealed the causative lesions of such cognitive and neuropsychiatric impairments, at least in humans. Thus, our case is the first to show an association between an abnormality on brain MRI and cognitive dysfunction, such as the frontal lobe atrophy with white matter lesions that might be associated with cognitive dysfunction in the proband’s mother. Whereas the primary pathology of SCA10 in humans is reportedly Purkinje cell loss [37], transgenic mice containing SCA10 pentanucleotide repeats showed neuronal loss in the cerebral cortex, hippocampus and pontine nuclei [38]. In addition to its expression in the brain, human ataxin-10 mRNA is expressed in a wide variety of tissues, including skeletal muscle, heart, liver and kidney [2,39]. Therefore, the changes in our case suggest that the pathological process is associated not only with the cerebellum but also with widespread regions of the brain, consistent with the presence of extracerebellar symptoms. It is possible that the cognitive impairment of the mother is due to the coincidental occurrence of dementia, as the proband did not show cognitive impairment. However, frontal lobe atrophy with white matter lesions associated with cognitive dysfunction may be a part of SCA10 pathology at a late stage of the disease. Certainly, frontal lobe atrophy with white matter lesions in our case was not reported previously in cases with SCA10. Although detailed neuropsychological examinations had not been conducted, the age of onset and the remarkable frontal lobe atrophy indicate the possibility of comorbidity, such as frontotemporal lobar degeneration (FTLD). While two mutations in one patient are rare, such cases have been reported [40, 41]. Thus, we investigated genes associated with FTLD. As a result, repeat-primed PCR revealed no expanded hexanucleotide repeats in the *C9orf72* gene in the proband or her mother. Likewise, we disclosed that they did not have *MAPT* and *PGRN* gene mutations. However, in Asia, the prevalence of patients with sporadic FTLD is more frequent than that with familial FTLD [42]; therefore, the possibility of comorbidity with FTLD cannot be excluded.

## Conclusions

Our cases represent the first report of SCA10 observed in a Japanese family. Similar to Mexican and Chinese SCA10 cases, our SCA10 patients presented with cerebellar ataxia and extracerebellar signs that included epilepsy and cognitive dysfunction. The presence of SCA10 in Japan is very significant with respect to the migration of the human race and suggests that we should perceive SCA10 as a differential diagnosis of autosomal dominant cerebellar ataxia in Japan, especially in cases with epilepsy.

## Supporting information

S1 Fig Fluorescent repeat-primed PCR analysis to assess *C9orf72* mutation in an SCA10 family. Fluorescent repeat-primed PCR analysis of the *C9orf72* gene revealed the presence of repeat expansion in the positive control but not in the proband (IV-1) or in her mother (III-3).
(TIF)
