# Supplementary figures and images for: First report of a Japanese family with spinocerebellar ataxia type 10: The second report from Asia after a report from China
Source: PLoS One. 2017 May 19;12(5):e0177955. doi: 10.1371/journal.pone.0177955 (PMC5438172; doi:10.1371/journal.pone.0177955)

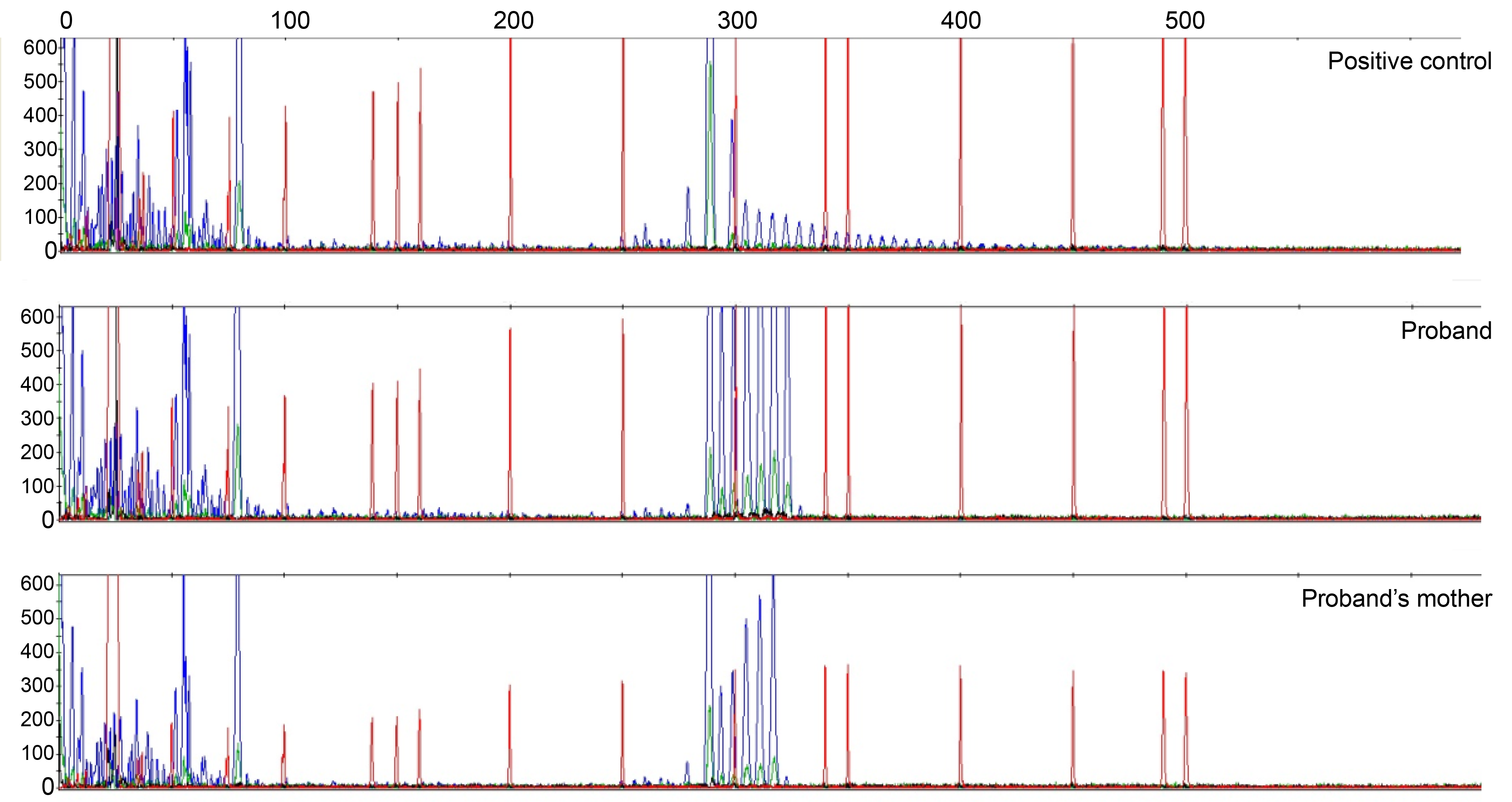

Supplement: S1 Fig — Fluorescent repeat-primed PCR analysis of the C9orf72 gene revealed the presence of repeat expansion in the positive control but not in the proband (IV-1) or in her mother (III-3). (TIF) [file pone.0177955.s001.tif]
